# Does second-look endoscopy reduce the bleeding after gastric endoscopic submucosal dissection for patients receiving antithrombotic therapy?

**DOI:** 10.1186/s12885-021-08679-7

**Published:** 2021-08-23

**Authors:** Takeshi Uozumi, Tetsuya Sumiyoshi, Yusuke Tomita, Kaho Tokuchi, Hiroya Sakano, Masahiro Yoshida, Ryoji Fujii, Takeyoshi Minagawa, Yutaka Okagawa, Kotaro Morita, Kei Yane, Hideyuki Ihara, Michiaki Hirayama, Hitoshi Kondo

**Affiliations:** Department of Gastroenterology, Tonan Hospital, Kita 4, Nishi 7, Chuo-ku, Sapporo, Hokkaido 060-0004 Japan

**Keywords:** Antithrombotic agents, Endoscopic submucosal dissection, Second-look endoscopy

## Abstract

**Background:**

In patients with average risk of bleeding, second-look endoscopy does not reportedly reduce bleeding after gastric endoscopic submucosal dissection. However, effectiveness of second-look endoscopy for patients with a high risk of bleeding, such as those who are taking antithrombotic agents, is unclear. Hence, this study aims to clarify the effectiveness of second-look endoscopy for patients with antithrombotic therapy.

**Methods:**

We studied 142 consecutive patients with 173 gastric epithelial neoplasms who were routinely taking antithrombotic agents and were treated by endoscopic submucosal dissection at Tonan Hospital between November 2013 and December 2019. They were classified into two groups: those with second-look endoscopy (SLE group, 69 patients with 85 lesions) and those without second-look endoscopy (non-SLE group, 73 patients with 88 lesions). The incidence of post-endoscopic submucosal dissection bleeding was compared between the SLE and non-SLE groups.

**Results:**

There were no statistical differences in the rate of patients undergoing single antiplatelet therapy, single anticoagulant therapy, and multiple therapy between the SLE and non-SLE groups (SLE group vs. non-SLE group; 32 [46.4%], 16 [23.2%], and 21 [30.4%] patients vs. 37 [50.7%], 20 [27.4%], and 16 [21.9%] patients, respectively; *p* = 0.50). Post-endoscopic submucosal dissection bleeding incidence was 21.7% (15/69) and 21.9% (16/73) in the SLE and non-SLE groups, respectively, and did not significantly differ between the two groups (*p* = 0.98).

**Conclusions:**

For patients taking antithrombotic agents, the incidence of post-endoscopic submucosal dissection bleeding was not reduced by second-look endoscopy.

## Background

Endoscopic submucosal dissection (ESD) has been the outstanding treatment for gastric epithelial neoplasms with a negligible risk of lymph node metastasis [1, 2]. However, the procedure can cause the formation of large iatrogenic ulcers, and it sometimes leads to problematic complications, such as bleeding and perforation [3]. Although the administration of proton pump inhibitor (PPI) and prophylactic coagulation after ESD were reportedly effective for preventing post-ESD bleeding [4–7], the incidence of bleeding after gastric ESD remains approximately 5%. Antithrombotic agents were reported as one of the risk factors for bleeding after gastric ESD, and the incidence of bleeding after gastric ESD for patients taking antithrombotic agents is higher (6.7–31.3%) than that in those who do not use them [8–10]. Bleeding after ESD may result in serious events, such as hypovolemic shock; thus, it is important to prevent it.

According to previous studies, rebleeding in patients with hemorrhagic peptic ulcer can be prevented by second-look endoscopy (SLE) after endoscopic hemostasis [11, 12]. Based on such studies, SLE was empirically performed after gastric ESD in many institutions [13]. However, three randomized controlled trials (RCTs) reported that routine SLE did not reduce bleeding after gastric ESD in patients with average risk of bleeding [14–16]. Among these RCTs, the SAFE trial, which was a Japanese multicenter prospective RCT, excluded patients with high risk of bleeding such as those who underwent antithrombotic therapy [14]. In the other two RCTs, the details of the antithrombotic agents were not described [15, 16]. Thus, to our knowledge, no studies have ever reported on the effectiveness of SLE for patients taking antithrombotic agents and the effectiveness of SLE is unclear.

In this study, we aimed to retrospectively assess whether SLE reduces bleeding after ESD in patients with high risk of bleeding, particularly focusing on those taking antithrombotic agents.

## Methods

### Patient selection

**Fig. 1:**
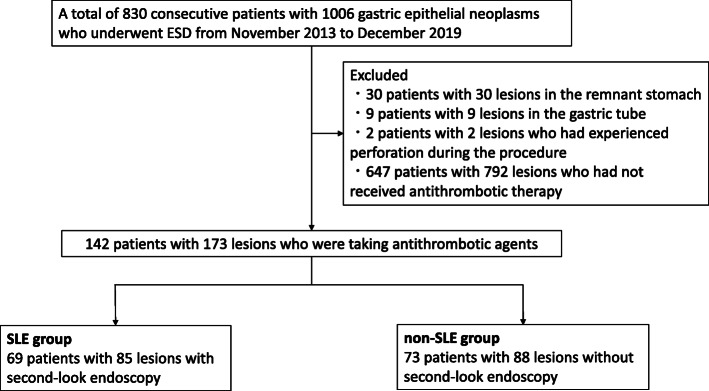
Patient selection flow diagram for the study

Medical records of patients who underwent ESD for gastric epithelial neoplasms, including early gastric cancers and adenomas, were retrospectively reviewed at Tonan Hospital from November 2013 to December 2019. These data included patient characteristics, endoscopic images, clinicopathological features, histopathological reports, and post-ESD complications. A total of 830 consecutive patients with 1006 gastric epithelial neoplasms underwent ESD. Among these patients, those with lesions arising from remnant stomach (*n* = 30) or gastric tube (*n* = 9), without antithrombotic therapy (*n* = 647), and with perforation during procedure (*n* = 2) were excluded from the study. Finally, 142 consecutive patients with 173 lesions, who were taking antithrombotic agents, were enrolled in this study (Fig. 1). SLE was empirically performed for all patients in our hospital; however, since January 2017, routine SLE was ceased in principle for all the patients including those with a high risk of bleeding after getting informed consent from them. These 142 patients were classified into the following two groups: SLE group (69 patients with 85 lesions) and non-SLE group (73 patients with 88 lesions). Prior to treatment, either the patient or their family provided written informed consents, and the study protocol was approved by the Institutional Review Board of Tonan Hospital and conducted in accordance with the Declaration of Helsinki.

### Patients

Data on patient characteristics, including age, sex, hypertension, diabetes, hyperlipidemia, ischemic heart disease, cerebrovascular disease, chronic kidney disease, treatment with antithrombotic agents, regular use of non-steroid anti-inflammatory drugs (NSAIDs), and treatment with gastric acid blockers, were collected. Antithrombic agents were classified as antiplatelet agents (aspirin, cilostazol, and thienopyridine) and anticoagulants (warfarin and direct oral anticoagulants [DOACs]). Gastric acid blockers were either PPIs or potassium-competitive acid blockers. The risk of post-ESD bleeding was evaluated for all patients according to the BEST-J score, which is a novel scoring system used to predict post-ESD bleeding in gastric epithelial neoplasms [17]. The risk of post-ESD bleeding was scored based on patient and lesion characteristics, and the total score was categorized as either low risk (post-ESD bleeding risk was 2.8%), intermediate risk (6.1%), high risk (11.4%), or very high risk (29.7%).

### Indications for ESD and ESD procedure

The indication for ESD included gastric adenoma and early gastric cancer that clinically fulfilled the absolute or expanded indication criteria according to the Japanese Gastric Cancer Treatment Guidelines of 2014 [18].

ESD was performed based on a standard procedure with a single-channel (GIF-Q260J; Olympus Medical Science) or a two-channel (GIF-2TQ260M; Olympus Medical Science) endoscope. In short, marking was made on the normal mucosa approximately 5 mm from the tumor margin. To lift the lesion, hyaluronic acid mixed with 10% glycerin solution in a 1:3 ratio with a small amount of epinephrine and indigo carmine was injected into the submucosal layer. After an initial incision was made using a needle knife (KD-1 L-1; Olympus Medical Science), an insulation-tipped knife 2 (KD-611 L; Olympus Medical Science) was used for making the circumferential incision and submucosal dissection. Intraoperatively, Coagrasper (FD-410LR; Olympus Medical Science) in soft coagulation mode was used to control bleeding. After completion of the ESD procedure, all exposed vessels on the iatrogenic ulcers were also coagulated using the Coagrasper in soft coagulation mode. All electrosurgical devices were used with an electrosurgical generator (VIO 300D; Erbe Elektromedizin). Based on the Japanese classification of gastric carcinoma, the resected specimens were examined pathologically with regard to tumor size, histological type, depth of invasion, lymphovascular invasion, and tumor involvement along the lateral and vertical margins [19].

### SLE

In this study, SLE was defined as a scheduled endoscopy that was performed on the day after ESD regardless of any suspicion of post-ESD bleeding. If the exposed vessels and/or active bleeding were observed on the post-ESD ulcer during SLE, hemostasis was performed with the Coagrasper in soft coagulation mode until they were sufficiently treated.

### Perioperative managements

Although 20 mg of esomeprazole was traditionally administered from the day of ESD to 8 weeks after the procedure, from August 2015, vonoprazan was administered from the day of ESD to 2 weeks after the procedure and then was switched to esomeprazole until 8 weeks after the ESD. The general condition of all patients was assessed via physical examination (including blood tests) the day after ESD; in the SLE group, the findings obtained during SLE were also assessed. The patients started drinking water on the day after ESD and resumed eating a soft diet 2 days after the procedure unless there were signs of ESD-related complications, such as perforation and post-ESD bleeding. Thereafter, the patients were discharged approximately on postoperative day 7 according to our clinical pathway.

### Management of antithrombotic agents

After confirming with the prescribing physician whether treatment with antithrombotic agents could be interrupted or not, ESD was performed in accordance with the Japanese Gastroenterological Endoscopy Society guideline [20]. Heparin replacement was done for patients taking antithrombotic agents until December 2016 at the physician’s discretion in accordance with the guideline. Since January 2017, heparin replacement was ceased for all patients and ESD was performed after the prothrombin time international normalized ratio (PT-INR) was confirmed to be within the therapeutic range when warfarin administration was continued. DOACs were continued until the day prior to ESD and ceased on the morning of ESD. When treatment with antithrombotic agents was interrupted, antithrombotic therapy was restarted the day after ESD in the non-SLE group and after the confirmation of no post-ESD bleeding during SLE in the SLE group.

Based on a previous report, in this study, antithrombotic agent interruption was defined as the cessation of antithrombotic agents including temporary replacement of other antithrombotic agents by heparin, aspirin, or cilostazol [17].

### Measured outcome parameters

Post-ESD bleeding was defined as hemorrhage with clinical symptoms, such as hematemesis, melena, and hemoglobin decrease of ≥2 g/dL compared to the patient’s latest data, which was confirmed by emergency endoscopy within 28 days after ESD, according to a previous report [14]. Subclinical bleeding during SLE was not counted as post-ESD bleeding. Post-ESD bleeding was classified into two phases: early and delayed bleeding. Early bleeding was defined as bleeding diagnosed within 24 h after ESD and delayed bleeding was defined as bleeding diagnosed later than the period that was defined as early bleeding. To evaluate the benefit of SLE, the incidence of post-ESD and delayed bleeding between the SLE and non-SLE groups were compared. The time-to-event curve for the absence of post-ESD bleeding between the two groups was evaluated. All patients were classified into three groups: single antiplatelet therapy, single anticoagulant therapy, and multiple antithrombotic therapy groups, and post-ESD bleeding and delayed bleeding rates of these groups were compared. Furthermore, all the patients were classified into four groups according to the BEST-J score: low risk group, intermediate group, high risk group, and very high risk group, and post-ESD and delayed bleeding rate of these groups were compared. The incidence of adverse events, such as blood transfusion and thromboembolism, was also compared between the SLE and non-SLE groups. The incidence of post-ESD bleeding in patients who received prophylactic coagulation during SLE was also evaluated.

### Statistical analysis

Fisher’s exact test, chi-square test, Welch’s *t* test, and student’s *t* test were used to appropriately analyze the significance of differences in the patient characteristics, clinicopathological findings, rate of post-ESD bleeding, rate of delayed bleedings, and rate of adverse events. Post-ESD bleeding rates from the day of procedure were calculated using the Kaplan–Meier method and compared using the log-rank test. A value of *p* < 0.05 was considered statistically significant.

## Results

### Patients and lesions

Table 1 shows the clinical characteristics of 142 consecutive patients with gastric epithelial neoplasms resected by ESD. Patients in the SLE group were younger than those in the non-SLE group (75.7 ± 7.1 years vs. 78.2 ± 6.2 years; *p* = 0.02). Sex and comorbidities, including ischemic heart disease, hypertension, diabetes mellitus, hyperlipidemia, cerebrovascular disease, and renal failure, were not significantly different between both groups. No patients received hemodialysis and regular NSAID administration. There were no significant differences in the rate of patients undergoing single antiplatelet therapy, single anticoagulant therapy, and multiple therapy between the SLE and non-SLE groups (SLE group vs. non-SLE group; 32 [46.4%], 16 [23.2%], and 21 [30.4%] patients vs. 37 [50.7%], 20 [27.4%], and 16 [21.9%] patients, respectively; *p* = 0.50). The SLE group had a significantly higher rate of patients experiencing antithrombotic agent interruption than the non-SLE group (55 patients [79.7%] vs. 41 patients [56.1%]; *p* < 0.05). Among the patients with interruption of antithrombotic therapy, 12 patients who received heparin replacement and 9 patients who received aspirin or cilostazol replacement were included in the SLE group and 10 patients who received aspirin or cilostazol replacement were included in the non-SLE group. The non-SLE group exhibited a significantly higher administration of vonoprazan than the SLE group (29 patients [42.0%] in the SLE group vs. 72 patients [98.6%] in the non-SLE group; *p* < 0.05). There were no statistical differences in the rate of patients who had multiple lesions between the two groups (11 patients [15.9%] in the SLE group vs. 14 patients [19.1%]; *p* = 0.61).

**Table 1:** Characteristics of patients

	SLE group (*n* = 69)	Non-SLE group (*n* = 73)	*p* values
Age (SD) (years)	75.7 ± 7.1	78.2 ± 6.2	0.02
Sex (Men/Women)	60 (87.0%) /9 (13.0%)	60 (82.2%) /13 (17.8%)	0.43
Comorbidities			
Hypertension	41 (59.4%)	53 (72.6%)	0.10
Diabetes mellitus	17 (24.6%)	26 (35.6%)	0.15
Hyperlipidemia	30 (43.5%)	35 (47.9%)	0.59
Ischemic heart disease	28 (40.6%)	29 (39.7%)	0.92
Cerebrovascular disease	28 (40.6%)	26 (35.6%)	0.54
Renal disease (eGFR < 30 mL/min/1.73m^2^)	1 (1.4%)	1 (1.4%)	0.97
Antithrombotic agent			0.50
Single antiplatelets	32 (46.4%)	37 (50.7%)	
Aspirin	15	20	
Cilostazol	6	5	
Thienopyridine	11	12	
Single anticoagulants	16 (23.2%)	20 (27.4%)	
Warfarin	7	7	
DOACs	9	13	
Multiple therapy	21 (30.4%)	16 (21.9%)	
Anticoagulant + antiplatelet	9	7	
DAPT	10	8	
Triplet therapy	2	1	
Interruption of antithrombotic agents	55 (79.7%)	41 (56.1%)	< 0.05
Heparin replacement	12	0	
Aspirin or cilostazol replacement	9	10	
Acid blocker			< 0.05
Esomeprazole	40 (58.0%)	1 (1.4%)	
Vonoprazan	29 (42.0%)	72 (98.6%)	
Multiple lesions	11 (15.9%)	14 (19.1%)	0.61

*SD standard deviation, *DOACs* direct oral anticoagulants, *DAPT* dual antiplatelet therapy

Table 2 presents the clinical characteristics of 173 lesions. No statistical differences were found in tumor location, tumor size, macroscopic type, histological type, ulcerative findings, histological depth, procedure time, specimen size, and en bloc resection between the two groups. The SLE group had a significantly lower number of lesions resected by an operator, whose experience was lower than 30 cases, than the non-SLE group (1 lesion [1.2%] vs. 11 lesions [11.4%]; *p* < 0.05).

**Table 2:** Characteristics of lesions

	SLE group (*n* = 85)	Non-SLE group (*n* = 88)	*p* values
Tumor location			0.50
Upper	10 (11.8%)	13 (14.8%)	
Middle	24 (28.2%)	30 (34.1%)	
Lower	51 (60.0%)	45 (51.1%)	
Tumor size (mm) (SD)	15.1 ± 12.5	15.2 ± 12.4	0.96
Macroscopic type			0.77
Protruding	38 (44.7%)	38 (43.1%)	
Flat/depressed	39 (45.9%)	44 (50.0%)	
Combined	8 (9.4%)	6 (6.8%)	
Histological type			0.30
Adenoma or differentiated type	82 (96.5%)	87 (98.9%)	
Undifferentiated type	3 (3.5%)	1 (1.1%)	
Ulcerative finding	6 (7.1%)	7 (8.0%)	0.82
Histological depth			0.95
Adenoma/m/SM1	79 (92.9%)	82 (93.1%)	
SM2	6 (7.1%)	6 (6.8%)	
Procedure time			0.97
≤ 60 min	61 (71.8%)	62 (70.4%)	
> 60 min ≤120 min	18 (21.2%)	20 (22.7%)	
> 120 min	6 (7.1%)	6 (6.8%)	
Specimen size (mm) (SD)	41.6 ± 14.9	42.0 ± 22.3	0.90
En bloc resection	85 (100%)	88 (100%)	
Operator experience of < 30 cases	1 (1.2%)	10 (11.4%)	< 0.05

*SD standard deviation

As to the risk classification of post-ESD bleeding scored by the BEST-J score, no statistical differences were observed in the low risk, intermediate risk, high risk, and very high risk (SLE group vs. non-SLE group; 11 [15.9%], 13 [18.8%], 31 [44.9%], and 14 [20.3%] patients vs. 6 [8.2%], 14 [19.2%], 37 [50.7%], and 16 [21.9%] patients, respectively; *p* = 0.56) (Table 3).

**Table 3:** Risk distribution of post-ESD bleeding scored by the BEST-J score

	SLE group (*n* = 69)	Non-SLE group (*n* = 73)	*p* values
BEST-J score			0.56
Low risk	11 (15.9%)	6 (8.2%)	
Intermediate risk	13 (18.8%)	14 (19.2%)	
High risk	31 (44.9%)	37 (50.7%)	
Very high risk	14 (20.3%)	16 (21.9%)	

### Post-ESD bleeding and delayed bleeding

**Fig. 2:**
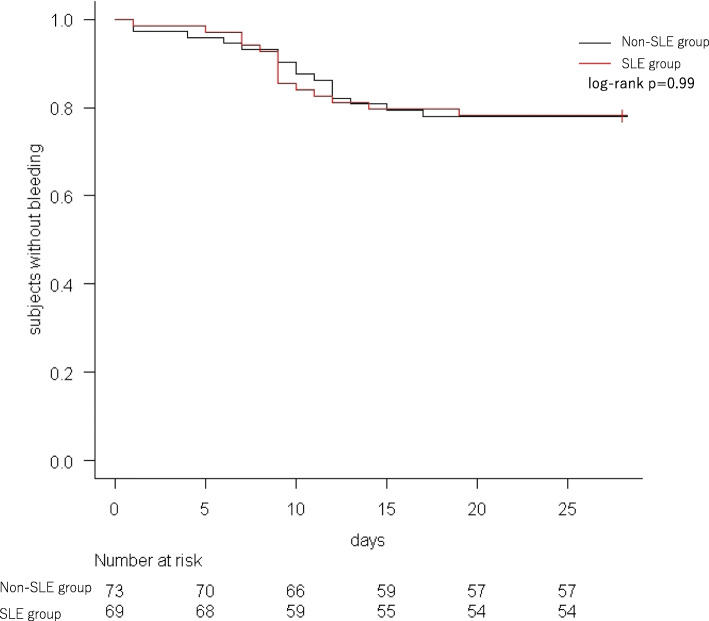
Time-to-event curve of patients without post-ESD bleeding

**Fig. 3:**
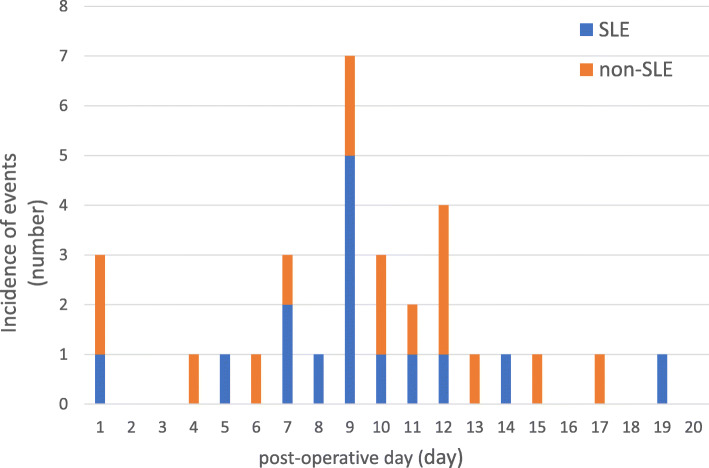
Timing of post-ESD bleeding

No significant difference was found in the incidence of post-ESD bleeding between the SLE group and non-SLE group (21.7% [15/69] vs. 21.9% [16/73]; *p* = 0.98) (Table 4), as well as the incidence of delayed bleeding (20.3% [14/69] vs. 19.2% [14/73]; *p* = 0.87). Likewise, the time-to-event curve for the absence of post-ESD bleeding showed no significant difference between the groups (SLE group vs. non-SLE group; 78.3, 95% CI: 66.6–86.3% vs. 78.1, 95% CI: 66.7–86.0%; *p* = 0.99) (Fig. 2). Post-ESD bleeding occurred within 2 weeks in 90.3% [28/31] of patients (Fig. 3). In addition, the incidence of post-ESD bleeding in patients undergoing single antiplatelet therapy (9.4% [3/32] vs. 10.8% [4/37]; *p* = 0.84), single anticoagulant therapy (6.3% [1/16] vs. 15.0% [3/20]; *p* = 0.41), and multiple therapy (52.4% [11/21] vs. 56.2% [9/16]; *p* = 0.82) was not significantly different between the SLE group and non-SLE group (Table 4); similar findings were obtained for the incidence of delayed bleeding.

**Table 4:** Details of post-ESD bleeding and delayed bleeding

	SLE group (*n* = 69)	Non-SLE group (*n* = 73)	*p* values
Post-ESD bleeding	15/69 (21.7%)	16/73 (21.9%)	0.98
Single antiplatelet	3/32 (9.4%)	4/37 (10.8%)	0.84
Single anticoagulant	1/16 (6.3%)	3/20 (15.0%)	0.41
Multiple therapy	11/21 (52.4%)	9/16 (56.2%)	0.82
Early bleeding	1/69 (1.4%)	2/73 (2.7%)	0.59
Delayed bleeding	14/69 (20.3%)	14/73 (19.2%)	0.87
Single antiplatelet	3/32 (9.4%)	3/37 (8.1%)	0.85
Single anticoagulant	1/16 (6.3%)	3/20 (15.0%)	0.41
Multiple therapy	10/21 (47.6%)	8/16 (50.0%)	0.89

The incidence of post-ESD bleeding in the low risk group of the BEST-J score (9.1% [1/11] vs. 16.7% [1/6]; *p* = 0.64), intermediate risk group (8.3% [1/13] vs. 7.1% [1/14]; *p* = 0.96), high risk group (19.4% [6/31] vs. 21.6% [8/37]; *p* = 0.82), and very high risk group (50% [7/14] vs. 37.5% [6/16]; *p* = 0.49) was not significantly different between the SLE group and non-SLE group (Table 5); similar findings were obtained for the incidence of delayed bleeding.

**Table 5:** The risk classification of Post-ESD bleeding scored by the BEST-J score

	SLE group (*n* = 69)	Non-SLE group (*n* = 73)	*p* value
BEST-J score			
Low risk	1/11 (9.1%)	1/6 (16.7%)	0.64
Intermediate risk	1/13 (8.3%)	1/14 (7.1%)	0.96
High risk	6/31 (19.4%)	8/37 (21.6%)	0.82
Very high risk	7/14 (50%)	6/16 (37.5%)	0.49

Subclinical bleeding was detected and hemostasis was performed in two cases during SLE. Prophylactic coagulation for exposed vessel was performed during SLE in 60patients in the SLE group. In the 62 patients who underwent prophylactic coagulation or hemostasis during SLE, the incidence of post-ESD bleeding was 22.6% (14/62) which was similar to that in the non-SLE group.

### Adverse events

The rate of bleeding that required transfusion was also not significantly different between the SLE group and non-SLE group (10.1% [7/69] vs. 11.0% [8/73]; *p* = 0.87). Thromboembolism did not occur in both groups.

## Discussion

Our study revealed that there was no significant difference in the incidence of post-ESD bleeding between the SLE and the non-SLE groups in patients taking antithrombotic agents.

Although all the patients underwent prophylactic coagulation after ESD completion and 89.8% (62/69) of patients in the SLE group received additional prophylactic coagulation or hemostasis during SLE, no significant difference was observed in the delayed bleeding rate between the two groups. Mochizuki et al., reported that prophylactic coagulation during SLE did not contribute to the reduction of post-ESD bleeding in patients with average risk of bleeding [14]. If prophylactic coagulation is adequately performed after ESD completion and a PPI is administered, additional prophylactic coagulation during SLE might not contribute to the reduction of post-ESD and delayed bleeding even in patients with high risk of bleeding. Moreover, the discontinuation of SLE after gastric ESD for patients with high risk of bleeding will reduce the economic burden on patients.

In our study, the non-SLE group exhibited a significantly higher administration of vonoprazan than the SLE group. Vonoprazan, a potassium-competitive acid blocker, has a stronger and longer antisecretory effect on H^+^/K^+^-ATPase than PPI [21]; however, on comparing esomeprazole and vonoprazan, no significant difference was noted in the incidence of post-ESD bleeding [22]. The difference in administration of vonoprazan or esomeprazole may not have affected the result of our study.

The incidence of post-ESD bleeding for patients with antithrombotic agents has been reported to be 6.7–31.3% [8–10]; these findings were similar those of our study. The reported risk factors for post-ESD bleeding are tumor size, tumor location, cardiopathy, chronic kidney disease, antithrombotic therapy, multiple antiplatelet therapy, and heparin replacement [23, 24]. There are several risk factors for post-ESD bleeding, which affect each other; therefore, the risk of post-ESD bleeding should be evaluated for each patient, considering comorbidities, tumor factor, and medication such as antithrombotic agents. Recently, Hatta et al. have reported the BEST-J score [17]. This prediction model consists of patient’s comorbidities, tumor factor, and medication, and comprehensively estimates the risk of post-ESD bleeding. There was no statistical difference between the SLE group and non-SLE group in the risk distribution of post-ESD bleeding scored by this prediction model, although the number of patients who underwent heparin replacement was significantly higher in the SLE group than in the non-SLE group. The incidence of post-ESD bleeding and delayed bleeding was not significantly different between the SLE group and non-SLE group regardless of the classification of antithrombotic agents and the risk distribution of the BEST-J score.

SLE might not reduce the incidence of post-ESD bleeding even in patients who received antithrombotic therapy; therefore, other endoscopic approaches are needed. In a nonrandomized control study including patients taking antithrombotic therapy, a tissue-shielding method using polyglycolic acid sheets and fibrin glue showed efficacy in the prevention of post-ESD bleeding (covering group, 5.8% vs control group, 20.8%; *p* = 0.04) [25]. Recently, Goto et al. have reported that hand-suturing method, which is a new technique for closing mucosal defects using absorbable barbed sutures and through-the-scope needle holder, might reduce the risk of post-ESD bleeding even in patients on antithrombotic therapy [26]. These promising methods require advanced endoscopic skills, and widespread adoption of these methods will take time.

This study has some limitations. First, it is a retrospective, single-center study with a small number of cases. Second, there were certain significant differences between the SLE and non-SLE groups with regard to patient characteristics and lesions, such as age, PPI administration, operator experience, interruption of antithrombotic therapy, and heparin replacement. Third, the risk of gastrointestinal bleeding varies among different DOACs [27], but the number of patients taking each kind of DOACs was too small to consider the effect of the bleeding. Although our study has these limitations, so far, no other study has estimated the effectiveness of SLE in patients with high risk of bleeding. Therefore, we believe that our study results are meaningful.

## Conclusions

In conclusion, SLE may not reduce the risk of post-ESD bleeding in patients with antithrombotic therapy. We hope that prospective randomized controlled trial will be conducted to clarify the effectiveness of SLE in patients under antithrombotic medication.

## Data Availability

The datasets used and/or analyzed during the current study are available from the corresponding author on reasonable request.

## Abbreviations

ESD: Endoscopic submucosal dissection
NSAID: Non-steroid anti-inflammatory drugs
PPI: Proton pump inhibitor
RCT: Randomized controlled trial
SLE: Second-look endoscopy
DOAC: Direct oral anticoagulants
